# Closing *Clostridium botulinum* Group I Genomes Using a Combination of Short- and Long-Reads

**DOI:** 10.3389/fmicb.2020.00239

**Published:** 2020-02-19

**Authors:** Narjol Gonzalez-Escalona, Shashi K. Sharma

**Affiliations:** Division of Microbiology, Office of Regulatory Science, Center for Food Safety and Applied Nutrition, Food and Drug Administration, College Park, MD, United States

**Keywords:** *Clostridium botulinum*, nanopore sequencing, MiSeq sequencing, foodborne pathogen, complete genomes, SNP phylogeny

## Abstract

*Clostridium botulinum* is a Gram-positive, spore-forming anaerobic bacterium that produces botulinum neurotoxin (BoNT). Closing their genomes provides information about their neurotoxin clusters’ arrangement(s) and their location (e.g., chromosome or plasmid) which cannot be assessed using draft genomes. Therefore, we tested the use of long-read sequencing (nanopore sequencing) in combination with short-read sequencing to close two toxin-producing strains. These genomes could be used by the Public Health Emergency Preparedness and Response staff during botulism outbreaks. The genomes of two toxin-producing *C. botulinum* strains, one from an environmental sample (83F_CFSAN034202) and the other from a clinical sample (CDC51232_CFSAN034200) were sequenced using MinION and MiSeq devices. The genomes, including the chromosomes and the plasmids, were closed by a combination of long-read and short-read sequencing. They belonged to different *C. botulinum* sequence types (STs), with 83F belonging to ST4 and CDC51232 to ST7. A whole genome single nucleotide polymorphism (SNP) analysis clustered these two strains with strains in lineage 2 (e.g., 6CDC297) and 4 (e.g., NCTC2916) from Group I, respectively. These two strains were also bivalent strains with the BoNTB and BoNTA4 clusters located in the larger plasmid for CDC51232, and the BoNTB and BoNTA1 clusters located both in the chromosome for 83F. Overall, this study showed the advantage of combining these two sequencing methods to obtain high quality closed *C. botulinum* genomes that could be used for SNP phylogenies (source tracking) as well as for fast identification of BoNT clusters and their gene arrangements.

## Introduction

*Clostridium botulinum* is a Gram-positive, spore-forming anaerobic bacterium that produces botulinum neurotoxin (BoNT) (Gill, 1982). Ingestion of the potent BoNT causes a serious paralytic illness known as botulism in humans and is a critical concern for food safety. BoNTs are produced by *C. botulinum* and some strains of other *Clostridium* species such as *butyricum*, *sporogenes*, *argentinense*, and *baratii* (Raphael et al., 2008, 2012). The *C. botulinum* serotypes are defined by the neurotoxins produced by these organisms which are serologically different and seven serotypes have been described, designated by the letters A through G (Shapiro et al., 1998), with type H still awaiting confirmation (Dover et al., 2014). The toxin types can be also subdivided into subserotypes based on their genetic diversity if they encoded a BoNT that differs in their amino acid sequence by at least 2.6% (Peck et al., 2017). Four of the seven serotypes (A, B, E, and F) have been linked with human botulism, with the majority of the cases due to serotypes A and B (Hill et al., 2007; Johnson and Montecucco, 2008). Botulinum toxins are listed as category A (the highest risk) threat agents by the US Centers for Disease Control and Prevention (CDC)^1^. However, BoNTs also have therapeutic applications, regardless of being dangerous biohazard agents, such as in the treatment of various muscle spasm disorders and for cosmetic purposes (Segura-Aguilar and Kostrzewa, 2006; Dressler, 2008; Pickett and Perrow, 2011).

*Clostridium botulinum* can be divided into four different groups (I–IV) on the basis of their 16S rDNA sequences, amplified fragment length polymorphism (AFLP) analysis (Hill et al., 2007), and metabolic profiles. Some authors have suggested the revision of the *C. botulinum* as a species based on whole genome sequencing (WGS) analyses, since the different groups are highly diverse and different from each other (Gonzalez-Escalona et al., 2014b; Smith et al., 2018). Members of Group I are proteolytic and produce BoNTs type A, B, and F. Members of Group II are non-proteolytic and produce BoNTs type B, E, and F. The majority of human cases of botulism are usually associated with members of Groups I and II (Hill et al., 2007; Jacobson et al., 2008a, b; Raphael et al., 2008; Sachdeva et al., 2010). In contrast, most cases of botulism caused by Group III have been recorded among animals. Group III strains produce BoNTs type C and D (Skarin and Segerman, 2011; Skarin et al., 2011). Finally, members of Group IV produce BoNT G. This last BoNT is the least-studied toxin and a clear implication in either human or animal botulism cases has not been established (Terilli et al., 2011). Genes responsible for BoNT production (*bont* genes) are located within a neurotoxin cluster, which contains genes arranged into two different conformations (Hill et al., 2009). One conformation contains hemagglutinin genes (*ha17*, *ha33*, and *ha70*) and the other contains *orfx* genes (*orfX*1, *orfX2*, and *orfX3*) (Hill et al., 2007; Smith et al., 2007; Jacobson et al., 2008a; Raphael et al., 2008). Depending on the conformation, the toxin gene clusters in subtype A1 strains are generally categorized as *ha* + *orfx-* or *ha- orfx* + BoNT, if hemagglutinin or *orfx* genes, respectively (Raphael et al., 2008). On the other hand, the toxin gene clusters in subtype B strains have been reported as only carrying hemagglutinin genes (*ha* + *orfx*- BoNT) (Hill et al., 2009).

With decreasing prices of WGS, more genomes of *C. botulinum* are produced yearly, from an initial 29 genomes at the beginning of 2014 to 294 genomes by May 2019^2^. WGS analyses provided valuable insights about the evolution and phylogeny of other foodborne bacterial pathogens causing outbreaks, including *Salmonella* Montevideo in 2010 (Bakker et al., 2011; Allard et al., 2012), *Vibrio cholerae* in Haiti in 2010 (Chin et al., 2011), *Escherichia coli* O104:H4 in Germany in 2011 (Rasko et al., 2011), and *Salmonella* Enteritidis in the United States in 2010 (Allard et al., 2013). In the case of *C. botulinum* group I the usefulness of genome-wide single nucleotide polymorphism (SNP) instead of traditional multilocus sequence typing (MLST) analysis for *C. botulinum* subtyping was highlighted in previous studies (Gonzalez-Escalona et al., 2014b; Raphael et al., 2014; Weigand et al., 2015).

While most of these genomes are in draft assemblies (Gonzalez-Escalona et al., 2014a, b; Raphael et al., 2014; Halpin et al., 2019), only 44 of the 294 genomes available at NCBI are completely closed. The BoNT operons analysis is difficult when using draft genomes and the location on the chromosome or plasmids is hard to define using these draft genomes.

Therefore, there is the need of developing methods for closing genomes that are available to most researchers, not only to those who can afford very expensive equipment such as PacBio.

In order to produce closed complete genomes, we tested the MinION nanopore sequencer, which has many attractive features compared to the gold standard PacBio, in conjunction with MiSeq Illumina sequencer for achieving completely closed *C. botulinum* genomes. To test this approach, we selected two bivalent toxin-producing *C. botulinum* strains (strains that carry two botulinum toxins) and sequenced them using both long-reads and short-reads in an effort to be prepared for future botulism outbreaks. The newly sequenced genomes herein were compared to other previously sequenced *C. botulinum* Group I genomes.

## Results and Discussion

### Whole Genome Assemblies

In this study, the closed genomes for the two bivalent *C. botulinum* strains were generated using a combination of long- and short-reads as described earlier (Gonzalez-Escalona et al., 2018). We generated 1.1 gigabase (Gb) pairs of sequence for 83F (300X genome average coverage) strain and 2.3 Gb for CDC15232 (630X genome average coverage). Each sample was run in a single flow cell. The MinION run for strain 83F produced a total of 239,626 reads with a read length N50 of 5 kb, while the run for CDC15232 produced a total of 963,433 reads with a read length N50 of 3 kb. Ninety-seven percent of the reads for 83F strain (232,387) and CDC15232 strain (932,465) were classified as belonging to *C. botulinum* by the WIMP workflow included in the Epi2me cloud service (Oxford Nanopore Technologies, Oxford, United Kingdom), confirming the identity and purity of these two strains. The number of short reads for each strain generated by MiSeq Illumina/Nextera DNA Flex were 2,920,033 (Q30 average length of 235 bp) for CDC15232 and 3,103,076 (Q30 average length of 236 bp) for 83F.

As explained in Section “Materials and Methods” and in Gonzalez-Escalona et al. (2018), after combining long- and short-reads, we were able to completely close both strains genomes including the chromosomes and their plasmids. Assembly statistics for each strain including number of contigs and sequence lengths are summarized in Table 1. The genome sizes for 83F and CDC51232 were 3.95 and 3.92 Mb, respectively. These genome sizes are similar to what has been described for *C. botulinum* Group I strains (Supplementary Table S1). Analysis of the resulting sequences showed the presence of two plasmids in each strain, differing in size and sequence (Table 1).

**TABLE 1:** Summary report of the *de novo* assembly of the two strains analyzed.

	83F (GC content%)	CDC51232 (GC content%)
Size chromosome (bp)	3.954,901 (28.2)	3,922,194 (23.2)
Size plasmids (bp)	57,676 (28.9) and 5926 (25.6)	270,024 (25.6) and 9953 (24.1)
# Contigs	3	3

Our goal was to use the long-read sequencing platform Oxford Nanopore in combination with Illumina MiSeq to produce high-quality, closed genomes for *C. botulinum* samples. We argue that this method could be considered preferable to using Pacific Biosciences (PacBio) Sequencers *RSII* or Sequel in our laboratory setting. We have previously compared these sequencing methods using *E. coli* (Gonzalez-Escalona et al., 2018, 2019). The MinION is affordable, deployable, and produces high-quality data, particularly when combined with the MiSeq. MiSeq sequencing produces a highly fragmented genome (e.g., 118 contigs for 83F) and the BoNT clusters will not be contained in single contigs, plus the location of those BoNT clusters (chromosome or plasmid) are hard to determine using only MiSeq data. This process will take weeks to obtain a result, compared to 4 days from DNA extraction to genome with the MiSeq and MinION combination approach. While PacBio is comprehensive in its ability to sequence the complete genome, it requires extensive space, time, and expertise. The MinION has been designed for portability and ease of use. The upfront cost of a MinION ($1000USD) is considerably more affordable than the PacBio ($450,000USD). PacBio requires significant starting material (1–5 μg), compared to approximately 400 ng for a nanopore flow cell. Furthermore, PacBio employs a size selection process that limits reads to ∼10 kb, which are smaller than that seen with the MinION (up to 1 Mb). The extended read length achieved by nanopore provides an easier and more exact scaffolding resulting in a more precise closed genome.

### Multilocus Sequence Typing Analysis of the Two *C. botulinum* Genomes

**FIGURE 1:**
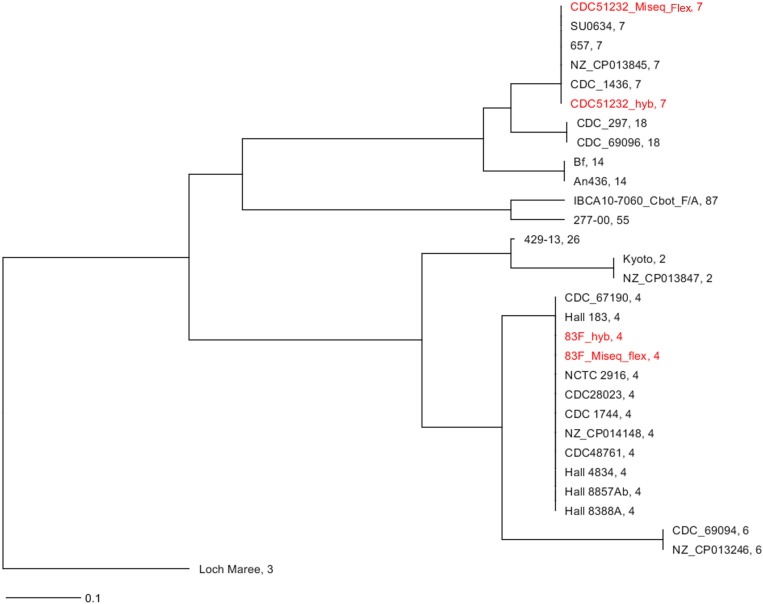
*In silico* MLST analysis of the strains sequenced in this study. NJ tree using seven loci for the two strains sequenced in this study and some closest relatives available at NCBI and rooted on A3 strain (CDC/A3). In red font are the strains sequenced in this study. The assembly generated from the MiSeq data was labeled as strain_ID_MiSeq_flex and the one generated by the combination of nanopore data (long-reads) and MiSeq (short-reads) was labeled as strain_ID_hyb. The ST for each strain assembly by either sequencing/assembly method was identical. The number after the name of the strain is the ST.

*In silico* MLST analysis^3^ showed that these strains belonged to known sequence types (STs), with CDC51232 being ST7 and 83F being ST4 (Table 1). The *C. botulinum* MLST database^4^ currently holds 23 strains in ST7 (all reported as serotype A, with varying sero subtype, e.g., strains Ba657 is Ba4). The number of strains reported for ST4 is less numerous with a total of 13 (all bivalent strains). A neighbor-joining (NJ) tree including selected strains with similar and/or related STs showed that these two strains were very different (Figure 1), and that both assemblies [Miseq-draft genome assembly obtained by using only MiSeq reads and hybrid-completely closed genome assembly obtained by using both short (MiSeq) and long (nanopore) reads] produced the same ST for each strain. This result indicates that the complete closed genome generated by our hybrid assembly using short- and long-reads produced a high-quality genome.

### Phylogenetic Relationship of the *C. botulinum* Strains by cgMLST Analysis

**FIGURE 2:**
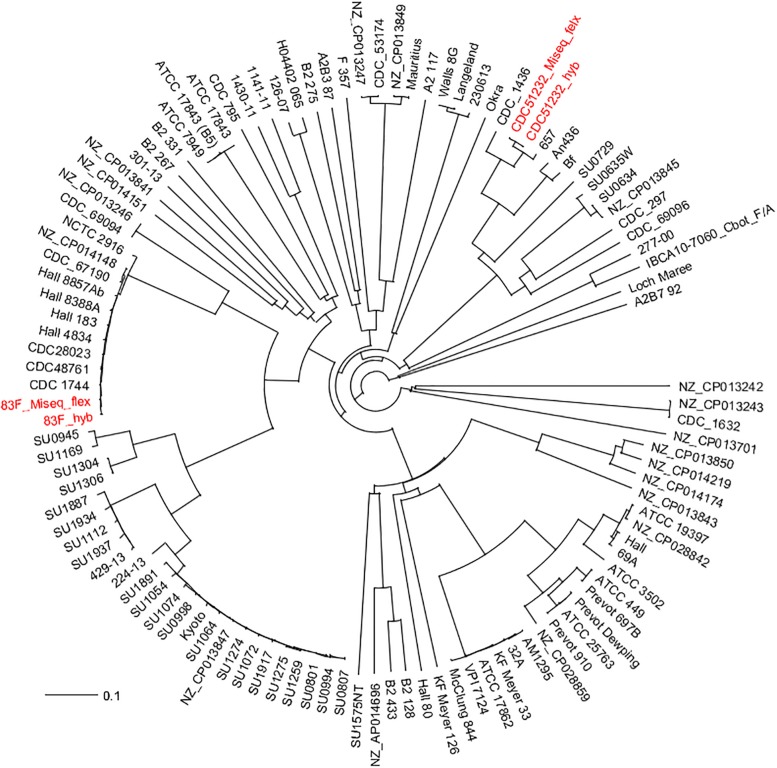
Initial cgMLST NJ phylogenetic tree based on gene differences on the 1159 shared genes (allele-based) of the two *C. botulinum* genomes generated in this study with the other 107 *C. botulinum* genomes downloaded from GenBank (Supplementary Table S1). In red fonts are the strains sequenced in this study. The assembly generated from the MiSeq data was labeled as strain_ID_MiSeq_flex and the one generated by the combination of nanopore data (long-reads) and MiSeq (short-reads) was labeled as strain_ID_hyb. Both assemblies from each strain were indistinguishable by the cgMLST analysis.

The phylogenetic relationship among the 111 *C. botulinum* genomes (107 downloaded from NCBI—Supplementary Table S1, and the two genomes generated in this study) was determined by a custom cgMLST analysis (Figure 2). The custom cgMLST scheme consisted in a total of 3277 genes [using the genome of *C. botulinum* A strain Hall (NC_009698) as reference], and only 1159 were present in each genome (core genome for this dataset) (Supplementary Table S2). The fact that most of the genomes were in draft stage (some genes might be truncated at either end of each contig and will not be identified by the software that requires to align 100% to call it) and that some genes might be unique to some strains could explain why only 1159 genes were shared among all 109 genomes. The genes that were randomly present were eliminated from the analysis. The first and fast phylogenetic analysis based on gene differences (allele-based) (NJ tree) among these 111 *C. botulinum* genomes (Figure 2) revealed an intricate evolutionary history with the existence of multiple, highly diverse genomic variants of strains. It also showed that the genomes generated by the short-read sequencing (MiSeq Illumina) were indistinguishable from the completely closed genome generated by a combination of both short- and long-read sequencing (hyb) for each strain.

**FIGURE 3:**
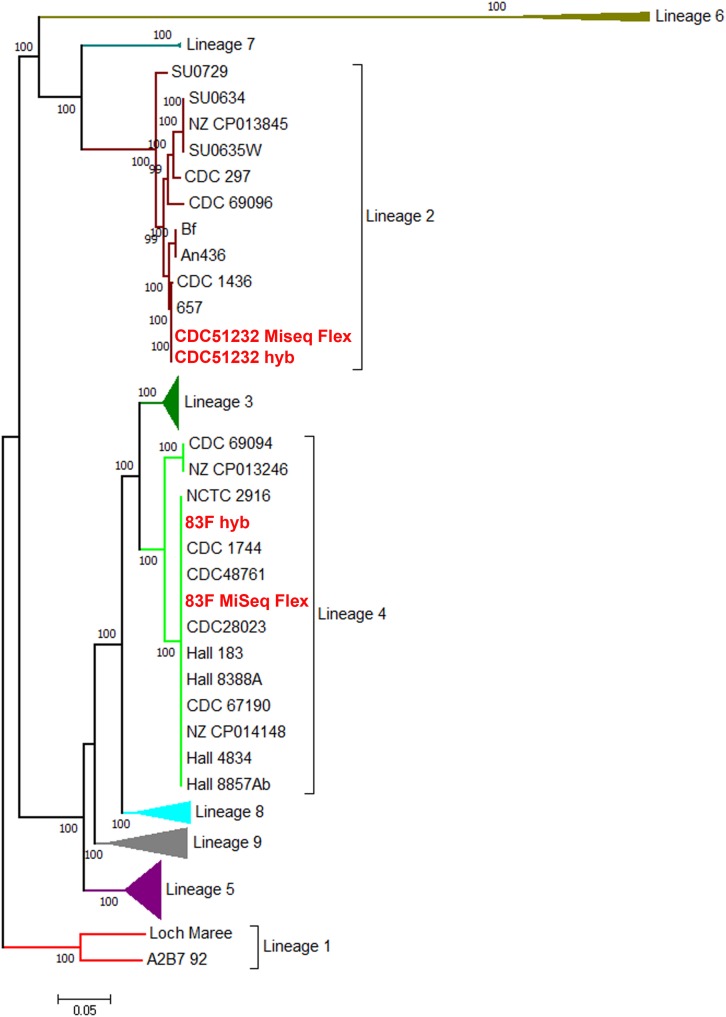
Maximum likelihood phylogeny of *C. botulinum* Group I from a 117,944 bp core SNP matrix. The evolutionary history was inferred by using the maximum likelihood method based on the Kimura two-parameter model (Kimura, 1980). The tree shown has most of the lineages compressed for visualization purposes. The original tree with the highest log likelihood is shown in Supplementary Figure S1. Strains are listed in Supplementary Table S1. Bootstrap supports above 50% are shown above the branches. In red fonts are the strains sequenced in this study. The tree is drawn to scale, with branch lengths measured in the number of substitutions per site. Evolutionary analyses were conducted in MEGA7 (Kumar et al., 2016).

A further whole genome SNP analysis from the 1159 core loci produced a matrix of 117,944 SNPs for these 111 *C. botulinum* genomes. The SNP matrix (Supplementary Data Sheet S1) was used to reconstruct the maximum likelihood phylogeny that showed that the two genomes sequenced in this study belonged to two different lineages (Figure 3). CDC51232 and 83F belonged to lineage 2 and 4, respectively, containing mostly bivalent strains, also observed in our previous studies (Gonzalez-Escalona et al., 2014b). This phylogenetic analysis further highlighted supported lineages that were not described in Gonzalez-Escalona et al. (2014b). In keeping with this previous publication, we have named the new lineages 6, 7, 8, and 9. The list of the strains belonging to each lineage can be found in Supplementary Table S3. Every lineage was represented by more than one strain. The existence of nine lineages in *C. botulinum* group I confirms the high diversity among members of this group (Figure 3). New lineages might be discovered when more strains of *C. botulinum* group I are sequenced, specially from other countries that are not represented in this genome set (Supplementary Table S1).

### *In silico* Toxin Cluster Analysis and Location

Although these two isolates were also bivalent *C. botulinum* strains, the location of the botulinum (BoNT) clusters differed between them. The neurotoxin cluster organization and location were determined for each strain (Table 2). In strain CDC51232, the BoNT clusters (BoNTB and BoNTA4) were located in the larger plasmid, while in 83F, these BoNT clusters (BoNTb5 and BoNTA1) were located in the chromosome (Table 2). Previous studies have identified six different A toxin types (Jacobson et al., 2008a; Raphael et al., 2008; Carter et al., 2011; Moritz et al., 2018). Regarding B toxin, there have been eight types reported (Halpin et al., 2019).

**TABLE 2:** Characteristics of the *C. botulinum* strains used in this study.

Isolate name	Serotype	Accession no.chromosome	Accession no.plasmids	SRAs	Cluster type	Toxin cluster location^a^	ST	Source
CDC51232	A, B	CP031097	**CP031096**, CP031095	SRR7530166, SRR7530167	*ha- orfX* + BoNTa4; ha + orfX- BoNTB5	plasmid	7	Clinical
83F	A, B	**CP031098**	CP031100, CP031099	SRR7532471, SRR7532470	*ha* + orfX- BoNTb5; ha- orfX + BoNTA1	Chr/oppA; Chr/arsC	4	Environmental

*^a^Location: Chr/arsC—chromosome in arsC operon, Chr/oppA—chromosome in oppA/brnQ operon. Accessions in bold indicate where the BoNT cluster are located. CFSAN numbers for CDC51232 are CFSAN034200 and CFSAN034202 for 83F. We provided CFSAN numbers to each strain that is sequenced in our laboratories for easy tracking in the system. CFSAN stands for Center for Food Safety and Applied Nutrition.*

**FIGURE 4:**
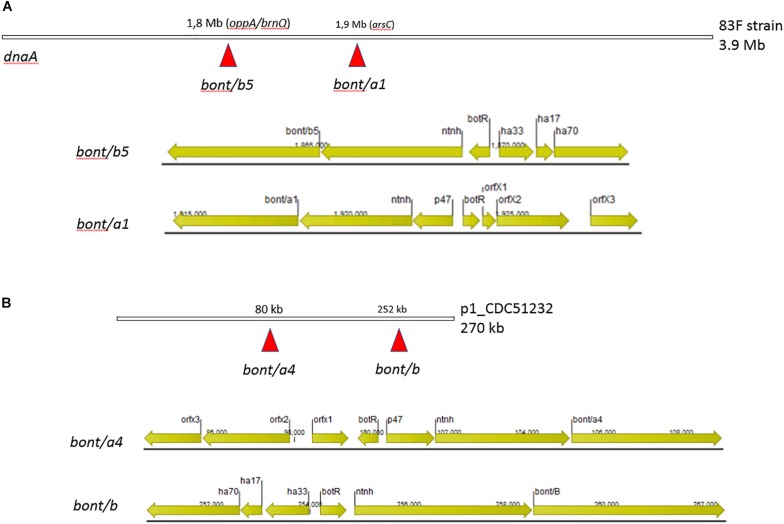
Schematic representation of the neurotoxin cluster organization and their relative location in the two strains sequenced in this study. The closed chromosomal genomes were rotated to start at the *dnaA* gene. **(A)** In strain 83F, the neurotoxin gene clusters were located at the chromosome inserted at operons *oppA/brnQ* and *arsC*. **(B)** In strain CDC51232, the neurotoxin gene clusters were located inserted at two separate locations on the large plasmid (270 kb).

In strain 83F, the neurotoxin gene clusters were located in the *oppA/brnQ* operon (*ha* + *orfX*-*bont/b5*) and in the *arsC* operon (*ha*- *orfX* + *bont/A1*) (Figure 4A). Hill et al. (2009) reported that type A toxin clusters in the chromosome are characteristically located at one of two sites: the *arsC* operon and the *oppA/brnQ* operon. Each neurotoxin cluster genes arrangement for this strain is shown in Figure 4A. The *bont/b* gene for this strain was 100% identical to another *bont/b5* gene (*bont/b* gene contains stop codons by mutations) present in CDC_67190 and Iwate2007 strains, which was reported as a silent B5 toxin producer in GenBank by Hill et al. (unpublished) and as a silent *bont/b* gene by Kenri et al. (2014). The ha- orfX + bont/A1 toxin gene cluster (14,794 bp) of 83F was 100% identical to the toxin gene cluster for two other strains with completely closed genomes reported in GenBank (CDC_69094-CP013246 and CDC_67190-CP014148 strains). Both of these latter strains have different genome sizes compared to 83F, with CDC_69094 being 4,089,027 bp and CDC_67190 a little smaller with 3,954,777 bp. They also belonged to the same *C. botulinum* lineage (lineage 4, Figure 3), but to different STs with CDC_69094 being ST6 and CDC_67190 to ST4. This re-enforces the transferable nature of these the toxin gene clusters among *C. botulinum* genomes as previously suggested (Hill et al., 2009; Skarin and Segerman, 2011; Gonzalez-Escalona et al., 2014b; Smith et al., 2015). Furthermore, CDC_67190 and 83F differed by only nine SNPs in a matrix of 117,944 SNPs, suggesting that these two strains are highly related, besides being ST4. Strain 83F (isolated in 1996) was also indistinguishable (0 SNPs) from strain CDC 1744 (LFOK01, isolated in 1977), and differed by two and five SNPs from strains CDC48761 (JFGG01, isolated in 1993) and CDC28023 (JFGM01, isolated in 1973), respectively. The genome of these last three strains are in draft status at GenBank. Interestingly, all these five highly related strains belonged to the same ST (ST4), while CDC_69094 belonged to ST6. Taking in consideration that the reported evolution rate for *Clostridium difficile*, a close relative of *C. botulinum*, is of approximately 1.1 SNP per year (Didelot et al., 2012; Eyre et al., 2013; Knight and Riley, 2016), we could assume a similar rate of evolution for *C. botulinum*, confirming the high relatedness of these five strains.

On the other hand, the neurotoxin gene clusters in CDC51232 were located in the larger plasmid (p1_CDC51232). They were organized as follows: *ha* + *orfX*-*bont/B5* and *ha*-*orfX* + *bont/a4* (Figure 4B). This larger plasmid was highly similar to a plasmid found in Ba4 strain 657 (pCLJ - CP001081) reported by Smith et al. in 2007 (Smith et al., 2007). CDC_51232 and Ba4 strain 657 differed by 24 SNPs in a matrix of 117,944 SNPs suggesting that these 2 strains are highly related. However, they were isolated from unrelated cases. Strain Ba4 657 was isolated from a case of infant botulism in Texas around 1977 (Edmond et al., 1977) while CDC_51232 was isolated in 2008. The number of SNPs differences increased to 48 when only the two strains were included in the cgMLST analysis. This was because they shared a larger number of loci (2755). This SNP differences re-enforced our previous assessment that they were highly related but belonging to different unrelated cases.

## Conclusion

This study reports the successful use of a combination of long and short read sequencing for obtaining high quality closed *C. botulinum* genomes (including their plasmids). The availability of this type of analysis for closing *C. botulinum* genomes allows for rapid close of genomes and to perform *in silico* analysis for determining the composition and location of the BoNT clusters as well as allowing for fast identification of the lineage and ST of the analyzed strains. This hybrid assembly approach also allows for determining not only the correct genome size but also the synteny of the chromosome and plasmids, and whether the analyzed strain carries any plasmid(s). Knowing the plasmid and its gene composition might be important in determining if it is needed for survival of that strain or provides other important clues about its origin or source. Generating these data is not possible when using MiSeq data alone. Both MiSeq and hybrid assembly using nanopore provides ST, SNP phylogeny, and lineage delineation. These high-quality closed genomes will be useful for SNP phylogenetic analysis for discrimination of *C. botulinum* strains and could be used by the Public Health Emergency Preparedness and Response staff during botulism outbreaks. It also demonstrates the utility for rapidly distinguishing *C. botulinum* strains containing identical toxin gene subtypes (A and B) and their toxin gene arrangements.

## Materials and Methods

### Bacterial Strains

The bacterial strains used in this study, 83F_CFSAN034202 and CDC51232_CFSAN034200, belonged to the FDA collection (Table 1). *C. botulinum* is a Biosafety level 2, select agent tier 1 bacterium and therefore was handled inside a select agent laboratory following CDC select agent guidelines. These *C. botulinum* strains were grown on trypticase-peptone-glucose-yeast (TPGY) extract and cooked meat medium (CMM) and incubated anaerobically at 37°C for 72 h. CMM cultures from each sample were inoculated by evenly spreading 100 μL on solid botulinum selective medium (BSM) and incubated anaerobically at 37°C for 72 h. Single colonies were picked up for DNA extraction.

### DNA Extraction and Quantification

Genomic DNA from each strain was isolated from overnight cultures using DNeasy Blood and Tissue Kit (QIAGEN, Valencia, CA, United States). The quality of the DNA was checked using a NanoDrop 1000 (Thermo Scientific, Rockford, IL, United States) and the concentration was determined using a Qubit double-stranded DNA HS assay kit and a Qubit fluorometer (Thermo Scientific) according to the manufacturer’s instructions.

### Whole Genome Sequencing, Contigs Assembly, and Annotation

The whole genomes were sequenced (WGS) and closed by using a combination of long-reads (using a MinION nanopore sequencer) and short-reads (using a MiSeq Illumina sequencer). The long-reads WGS for each strain were generated through MinION sequencing (Oxford Nanopore Technologies, Oxford, United Kingdom). The sequencing libraries were prepared using the rapid sequencing kit RAD004 and run in FLO-MIN106 (R9.4.1) flow cells, according to the manufacturer’s instructions, for 48 h, at 300–630X average coverage. The sequencing library contained DNA fragmented randomly by a transposase present in the Fragmentation Mix of the RAD004 kit, rendering fragments > 30 kb. The run was live base called using Albacore v2.2.7 included in the MinKNOW 2.2 (v18.07.2) software (Oxford Nanopore). The initial classification of the reads for each run was done using the “What’s in my pot” (WIMP) workflow contained in the Epi2me cloud service (Oxford Nanopore Technologies, Oxford, United Kingdom). That workflow allows for taxonomic classification of the reads generated by the MinION sequencing in real time. The short-read WGS for each strain was generated using MiSeq Illumina sequencing with the MiSeq V3 kit using 2 × 250 bp paired-end chemistry, (Illumina, San Diego, CA, United States) according to manufacturer’s instructions, at 160–180X average coverage. The libraries for the MiSeq were constructed using 100 ng of genomic DNA using Nextera DNA Flex kit (Illumina), according to the manufacturer’s instructions. The genomes for each strain were obtained by *de novo* assembly, using nanopore data and default settings within CANU program v1.6 (Koren et al., 2017). A second assembly was generated using a SPAdes v3.12.0 (Bankevich et al., 2012) hybrid assembly (with default settings) using both nanopore and MiSeq data generated for each strain. The final assembly (FA) was generated by comparing the SPAdes hybrid and CANU assemblies using Mauve (Darling et al., 2004) and filling in the missing regions in the SPAdes assembly with the CANU assembly. The FA sequences were annotated using the NCBI Prokaryotic Genomes Automatic Annotation Pipeline (PGAAP^5^) (Tatusova et al., 2016).

### *In silico* MLST Phylogenetic Analysis

Multilocus sequence typing analysis was conducted using seven loci (*aroE*, *mdh*, *aceK*, *oppB*, *rpoB*, *recA*, and *hsp*) described previously for *C. botulinum* strains (Jacobson et al., 2008b). The sequence for each allele and the ST was obtained by *in silico* querying the MLST database for *C. botulinum*^4^. The STs are defined by the combination of the seven loci profile. We used Nei’s DNA distance method (Nei et al., 1983) for calculating the matrix of genetic distance, taking into consideration only the number of same/different alleles in the seven loci. An NJ tree using the appropriate genetic distances was built after the MLST analysis. The tree was rooted with *C. botulinum* subtype A3 strain Loch Maree.

### Whole Genome Phylogenetic Analysis

We analyzed 107 *C. botulinum* genomes publicly available at GenBank belonging to *C. botulinum* Group I (Supplementary Table S1) alongside our two newly sequenced genomes (hybrid and Illumina only assemblies), comprising a dataset of 111 draft and complete genomes. The ∼180 other genomes of *C. botulinum* at NCBI belong to the other *C. botulinum* groups and were not included in this comparison since they are very divergent. The phylogenetic relationship of the strains was assessed by a core genome MLST (cgMLST) analysis using Ridom SeqSphere + software v6.0.0 (Ridom GmbH, Münster, Germany). *C. botulinum* A strain Hall genome (NC_009698) was used as the reference for the cgMLST. The genome of this strain has 3332 genes, of which 2439 genes (core genes) were present in the nine of the completely closed *C. botulinum* genomes used for comparison: A2 strain Kyoto (NC_012563.1), A3 strain Loch Maree (NC_010520.1), A strain ATCC 19397 (NC_009697.1), A strain ATCC 3502 (NC_009495.1), B1 strain Okra (NC_010516.1), Ba4 strain 657 (NC_012658.1), F strain 230613 (NC_017297.1), F strain Langeland (NC_009699.1), and strain H04402 065 (NC_017299.1). While 838 were found in some of the compared genomes, the remaining 55 genes were eliminated from the analysis since they were paralogous or pseudogenes. Therefore, a total of 3277 genes were used as templates for the analysis of the *C. botulinum* strains from this study. After eliminating loci that were missing from the genome of any strain used in our analyses, we performed a cgMLST analysis. These remaining loci (1159) were considered the core genome shared by the analyzed strains (Supplementary Table S2). We used Nei’s DNA distance method (Nei et al., 1983) for calculating the matrix of genetic distance, taking into consideration only the number of same/different alleles in the core genes. An NJ tree using the appropriate genetic distances was built after the cgMLST analysis. cgMLST uses the allele number of each loci for determining the genetic distance and builds the phylogenetic tree. The use of allele numbers reduces the influence of recombination in the dataset studied and allows for fast clustering determination of genomes. The SNPs were extracted from the core loci and the SNP matrix was used to reconstruct the maximum likelihood phylogeny using Mega software v7 (Kumar et al., 2016) using the Kimura two-parameter model to estimate the genetic distances. The statistical support of the nodes in the ML tree was assessed by 500 bootstrap re-sampling. The use of SNPs allowed for determining a true phylogeny and to find informative SNPs in the dataset. The tree was rooted with *C. botulinum* subtype A3 strain Loch Maree.

### *In silico* Toxin Cluster Analysis and Location

In order to assess the organization of the toxin gene cluster and its insertion site in the two strains, the annotated chromosome (83F) or plasmid (CDC51232) containing the neurotoxin clusters were perused for the presence of BoNTs. The section containing the BoNTs was extracted and visualized with CLC genomics workbench (QIAGEN) and the annotation was manually corrected to display the correct nomenclature for the genes present in the neurotoxin clusters.

## Data Availability Statement

The closed genome sequences of the *C. botulinum* strains were deposited in GenBank under accession numbers listed in Table 2.

## Author Contributions

NG-E and SS conceived and designed the experiments, contributed reagents, materials, and analysis tools, and wrote the manuscript. NG-E analyzed the data.

## Conflict of Interest

The authors declare that the research was conducted in the absence of any commercial or financial relationships that could be construed as a potential conflict of interest.

## Footnotes

1 https://emergency.cdc.gov/agent/agentlist-category.asp

2 https://www.ncbi.nlm.nih.gov/pathogens/isolates/#/search/taxgroup_name:%22Clostridium%20botulinum%22

3 https://pubmlst.org/bigsdb?db=pubmlst_cbotulinum_seqdef&page=sequenceQuery

4 http://pubmlst.org/cbotulinum/

5 http://www.ncbi.nlm.nih.gov/genome/annotation_prok

## Supplementary Material

FIGURE S1 Original maximum likelihood phylogeny of *C. botulinum* Group I from a 117,944 bp core SNP matrix shown in Figure 3 without the lineages compressed. The evolutionary history was inferred by using the maximum likelihood method based on the Kimura two-parameter model (Kimura, 1980). The original tree with the highest log likelihood is shown in. Strains are listed in Supplementary Table S1. Bootstrap supports above 50% are shown above the branches. In red fonts are the strains sequenced in this study. The tree is drawn to scale, with branch lengths measured in the number of substitutions per site. Evolutionary analyses were conducted in MEGA7 (Kumar et al., 2016).

TABLE S1 *C. botulinum* group I genomes used for phylogenetic analysis in this study.

TABLE S2 *C. botulinum* group I core genes used for the cgMLST analysis of the 111 strains in this study (1159 loci). The allele for each strain is also shown. Allele 1 corresponds to reference *C. botulinum* A strain Hall (NC_009698).

TABLE S3 *C. botulinum* group I genomes organized per lineages (Figure 3).

DATA SHEET S1 Matrix of 117,944 SNPs extracted from 1159 core loci from the 111 *C. botulinum* genomes for performing the whole genome single nucleotide polymorphism (SNP) analysis and used to reconstruct the maximum likelihood phylogeny for those 111 genomes.
