# Supplementary figures and images for: Closing Clostridium botulinum Group I Genomes Using a Combination of Short- and Long-Reads
Source: Front Microbiol. 2020 Feb 19;11:239. doi: 10.3389/fmicb.2020.00239 (PMC7050642; doi:10.3389/fmicb.2020.00239)

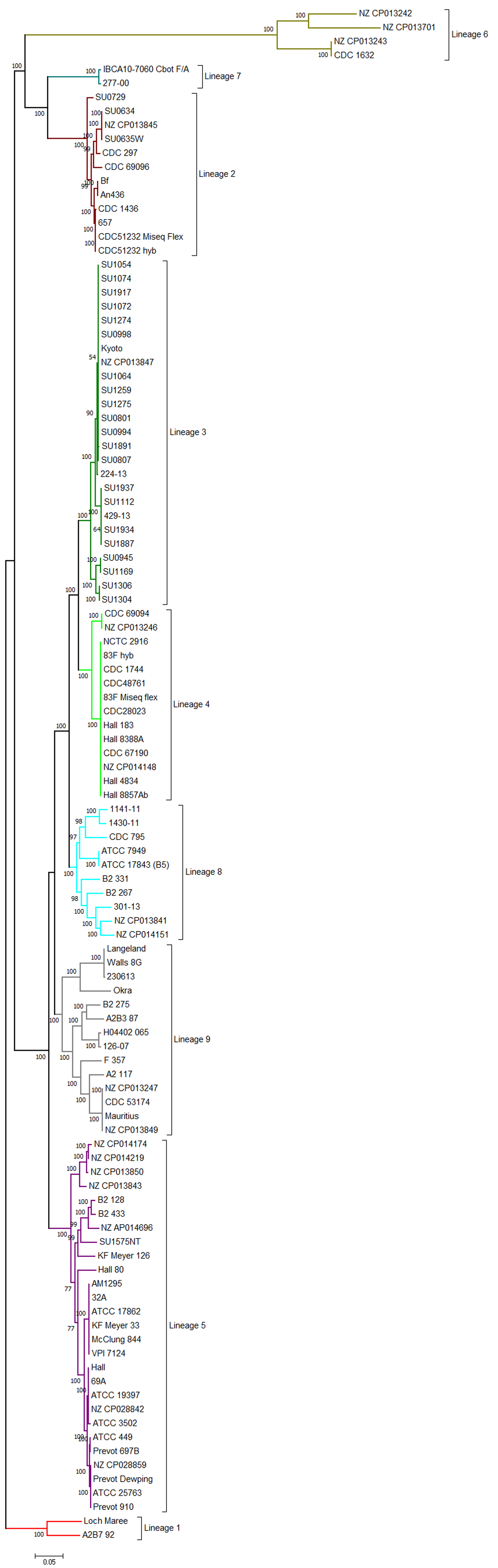

Supplement: FIGURE S1 — Original maximum likelihood phylogeny of C. botulinum Group I from a 117,944 bp core SNP matrix shown in Figure 3 without the lineages compressed. The evolutionary history was inferred by using the maximum likelihood method based on the Kimura two-parameter model (Kimura, 1980). The original tree with the highest log likelihood is shown in. Strains are listed in Supplementary Table S1. Bootstrap supports above 50% are shown above the branches. In red fonts are the strains sequenced in this study. The tree is drawn to scale, with branch lengths measured in the number of substitutions per site. Evolutionary analyses were conducted in MEGA7 (Kumar et al., 2016). [file Image_1.TIF]
